# Prognosis of patients with prostate cancer and middle range prostate - specific antigen levels of 20 – 100 ng / mL

**DOI:** 10.1590/S1677-5538.IBJU.2018.0143

**Published:** 2019

**Authors:** Hiroaki Iwamoto, Kouji Izumi, Yoshifumi Kadono, Atsushi Mizokami

**Affiliations:** 1 Department of Integrative Cancer Therapy and Urology, Kanazawa University Graduate School of Medical Science, Kanazawa, Japan

**Keywords:** Biomarkers, Prognosis, Prostatic Neoplasms

## Abstract

**Introduction:**

Prostate - specific antigen (PSA) is a useful biomarker for detection of prostate cancer (PCa) and for risk classification in addition to TNM classification and Gleason score (GS). We reported the role of PSA in patients with low (< 20 ng / mL) and extremely high (≥ 100 ng / mL) PSA levels. However, it is unclear whether a correlation exists between middle range PSA levels (20 – 100 ng / mL) at diagnosis and prognosis.

**Materials and Methods:**

Between January 2000 and December 2014, 1873 patients underwent prostate biopsy at Kanazawa University Hospital. Of 802 patients who were diagnosed with PCa, 148 patients with middle range PSA levels (20 – 100 ng / mL) were retrospectively analyzed.

**Results:**

The percentage of patients with T3 – 4 consistently increased as PSA levels increased from 20 to 100 ng / mL. Although the percentage of patients with GS ≥ 8 or metastases increased as PSA levels increased up to approximately 70 ng / mL, there was no significant increase between 70 and 100 ng / mL. PCa - specific and castration - resistant PCa - free survivals were adversely associated with PSA levels up to 70 ng / mL, but not between 70 and 100 ng / mL.

**Conclusion:**

PSA is a useful biomarker for predicting prognosis at levels between 20 and 70 ng / mL. However, PSA cannot be used as a prognostic factor in patients with PCa and PSA levels ≥ 70 ng / mL. When the PSA level reaches approximately 70 ng / mL, prognosis might bottom and reach a plateau.

## INTRODUCTION

Prostate - specific antigen (PSA) is a useful biomarker for the detection of prostate cancer (PCa) and risk classification in addition to TNM classification and Gleason score (GS) (1). D’Amico's risk classification, the most widely used localized PCa risk classification, consists of a diagnostic PSA level, high biopsy GS, and clinical T stage and is helpful in predicting prognosis (2). We previously showed that patients with PCa and a low level PSA (< 3.5 ng / mL) tend to be in more advanced stages than those with PSA levels between 3.5 ng / mL and 10 ng / mL and that PSA could no longer be used as a prognostic factor in patients with PCa and extremely high PSA levels (> 100 ng / mL) (3, 4). PSA > 20 ng / mL is defined as high - risk for PCa as per D’Amico's risk classification, and it is reasonable to assume that patients with a PSA level near 100 ng / mL have more advanced disease and a worse prognosis than those with a PSA level near 20 ng / mL. However, it is still unclear whether the PSA level itself is actually a prognostic factor in patients with PCa and middle range PSA levels (20 – 100 ng / mL). In this study, we retrospectively examined whether PSA could predict patient prognosis in patients with PCa and middle range PSA levels (20 – 100 ng / mL) and clarified factors that affect the survival of such patients.

## MATERIALS AND METHODS

Between January 2000 and December 2014, 1873 patients underwent prostate biopsy at Kanazawa University Hospital. Of 802 patients diagnosed with PCa, 148 patients with middle range PSA levels (20 – 100 ng / mL) were retrospectively analyzed. Demographic, surgical, pathological, and follow-up data were collected from medical charts and retrospectively analyzed. The follow-up was stopped on January 31, 2016. During the study period, all therapeutic decisions were left to the discretion of each attending physician. Patient distribution of every 10 ng / mL of PSA ranging up to 100 ng / mL was analyzed. Statistical analyses were performed using the commercially available software Prism (GraphPad, San Diego, CA). Comparisons of tendencies among different groups were performed by chi - square test for trends. Overall survival (OS), PCa - specific survival (PCaSS), and castration - resistant PCa (CRPC) - free survival (CFS) rates were estimated using the Kaplan - Meier method. Log - rank test for trend was used for comparison of survival distributions. In all analyses, p < 0.05 was taken to indicate statistical significance. This study was approved by Medical Ethics Committee of Kanazawa University (No. 2016 – 328).

## RESULTS

Patient characteristics are shown in Table-1. Median patient age at diagnosis of PCa was 73 (range: 53 – 89) years. Median follow-up was 58.4 (range: 0.03 – 176.4) months. Twenty - six patients had metastasis and 14 patients developed CRPC. All patients underwent androgen - deprivation therapy (ADT), except for six patients who were treated with radical prostatectomy or radiotherapy alone. Sixty - three patients underwent both ADT and radiotherapy. Eight patients underwent both ADT and radical prostatectomy.

**Table 1:** Patient characteristics.

PSA ng/mL			20≤ <30	30≤ <40	40≤ <50	50≤ <60	60≤ <70	70≤ <80	80≤ <90	90≤ <100	Total
n			50	28	20	16	7	10	7	10	148
Median age,			71.5	78	69	75.5	81	71.5	68	77	73
year (range)			(55-86)	(53-89)	(56-81)	(64-85)	(56-83)	(60-83)	(59-86)	(65-85)	(53-89)
**T**											
	1, 2		37	18	11	8	3	4	3	3	87
	3, 4		13	10	9	8	4	6	4	7	61
**NM** †										
		N0 and M0	48	23	15	10	5	6	5	9	121
		N1 or M1	2	5	5	6	2	3	2	1	26
**GS** †										
		<7	9	1	2	3	0	0	0	3	18
		7	23	13	8	4	2	5	4	3	62
		>7	18	13	10	9	5	5	3	4	67
**CRPC dev**										
		Yes	2	1	2	1	3	2	2	1	14
		No	48	27	18	15	4	8	5	9	134
Median FU,			65.3	24.6	68.2	24.7	65.1	90.8	76.1	63.8	58.4
month (range)			(12.4-184.5)	(0.2-188.3)	(0.4-179.2)	(0.03-187.1)	(20.7-121.2)	(9.8-152.9)	(54.4-109.5)	(16.5-153.7)	(0.03-176.4)

**PSA** = prostate-specific antigen;

**GS** = Gleason score;

**CRPC dev** = castration-resistant prostate cancer development;

**FU** = follow-up.

† Data not available in one patient each.

**Figure 1:**
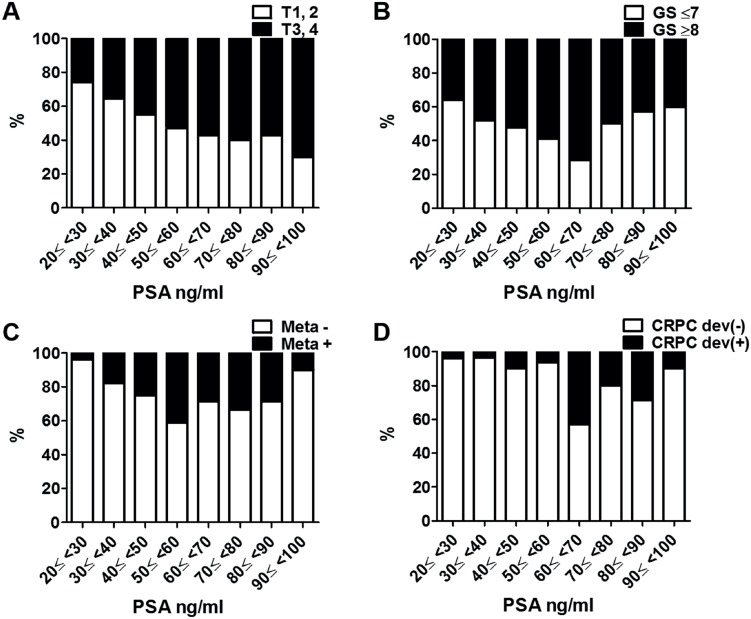
The distribution of clinical factors in every 10 ng / mL across the range of PSA levels. (A) The distribution of T stage indicates that the percentage of patients with T3 – 4 significantly increased between 20 and 100 ng / mL (p < 0.001). (B) The distribution of GS indicates that the percentage of GS ≥ 8 constantly increased between PSA levels 20 and 70 ng / mL (p = 0.023), whereas a significant increase was not observed between PSA levels 70 and 100 ng / mL (p = 0.220). (C) The distribution of the presence of metastasis (N or M) indicates that the percentage of metastasis (+) significantly increased between PSA levels 20 and 70 ng / mL (p < 0.001), whereas a significant increase was not observed between PSA levels 70 and 100 ng / mL (p = 0.120). (D) The distribution of patients with CRPC development (dev) (+) indicates that the presence of CRPC dev (+) patients significantly increased between PSA levels 20 and 70 ng / mL (p = 0.023), whereas no significant increase was observed between PSA levels 70 and 100 ng / mL (p = 0.220).

Figure-1 shows the relationship between the PSA level and T stage / GS / metastasis / CRPC development. The distribution in every 10 ng / mL across the range of PSA levels was analyzed. The percentage of patients with T3 and 4 significantly increased between PSA levels 20 and 100 ng / mL (Figure-1A). Although the percentage of patients with GS ≥ 8 significantly increased between PSA levels 20 and 70 ng / mL, a significant increase was not observed between PSA levels 70 and 100 ng / mL (Figure-1B). Also, the percentage of patients with metastases significantly increased between PSA levels 20 and 60 ng / mL, but a significant increase was not observed between PSA levels 60 and 100 ng / mL (Figure-1C). Furthermore, the percentage of patients with CRPC development (+) significantly increased between PSA levels 20 and 70 ng / mL; however, a significant increase was not observed between PSA levels 70 and 100 ng / mL (Figure-1D).

**Figure 2:**
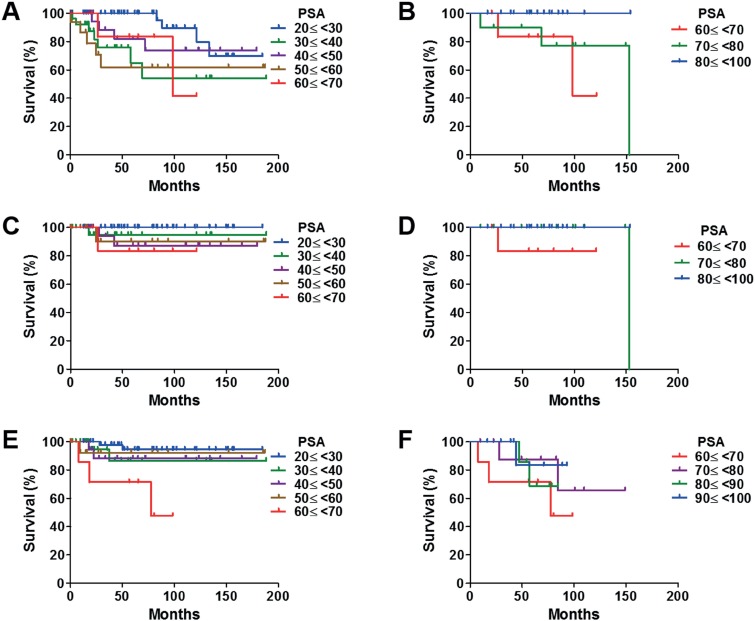
Kaplan - Meier analyses of OS, PCaSS, and CFS in patients based on PSA. (A) Higher PSA levels shortened OS significantly between 20 and 70 ng / mL (p = 0.029). (B) However, PSA level was no longer a significant factor for OS between 70 and 100 ng / mL, rather higher PSA prolonged OS (p = 0.040). (C) An inverse correlation between PSA levels and PCaSS was observed, where higher PSA levels significantly shortened PCaSS between 20 and 70 ng / mL (p = 0.026). (D) However, the PSA level was no longer a significant factor for PCaSS between 70 and 100 ng / mL (p = 0.083). (E) Higher PSA levels significantly reduced CFS between 20 and 70 ng / mL (p = 0.014). (F) However, the PSA level was no longer a significant factor for CFS between 70 and 100 ng / mL (p = 0.292).

In all patients, the 5 - and 10 - year OS rates were 87.0% and 77.5%, respectively. Although high T stage and GS significantly shortened OS, the presence of metastasis was not a significant factor for shortening OS. PSA shortened OS significantly as PSA levels increase between 20 and 70 ng / mL, whereas PSA extended OS significantly when PSA levels increased between 70 and 100 ng / mL (Figures 2A and B).

In all patients, 5 - and 10 - year PCaSS rates were 95.5%. High T stage and GS and the presence of metastasis significantly shortened PCaSS. Although PSA shortened PCaSS significantly when PSA levels were between 20 and 70 ng / mL, there was no significant difference in PCaSS rates when PSA levels were between 70 and 100 ng / mL (Figures 2C and D).

In all patients, the 5 - and 10 - year CFS rates were 87.9% and 84.1%, respectively. High T stage and GS and the presence of metastasis significantly shortened CFS. Although PSA shortened CFS significantly when PSA levels were between 20 and 70 ng / mL, there was no significant difference in PCaSS or in OS when PSA levels were between 70 and 100 ng / mL (Figures 2E and F).

## DISCUSSION

Previously, we reported that patients with PCa and a PSA level between 10 and 20 ng / mL at diagnosis had a more advanced stage of cancer than those with PCa and a PSA level between 3.5 and 10 ng / mL, whereas patients with PCa and a PSA level < 3.5 ng / mL at diagnosis potentially had a more advanced stage of cancer than those with PCa and a PSA level between 3.5 and 10 ng / mL (3). On the other hand, patients with PCa and a PSA level ≥ 100 ng / mL had a similar prognosis, suggesting that PSA alone is no longer useful as a prognostic marker when the initial PSA level is ≥ 100 ng / mL (4). Although it is believed that a higher PSA level indicates poor prognosis in patients with PCa, the prognosis of patients with PCa and a PSA level between 20 and 100 ng / mL has, so far, not been investigated in detail.

**Figure 3:**
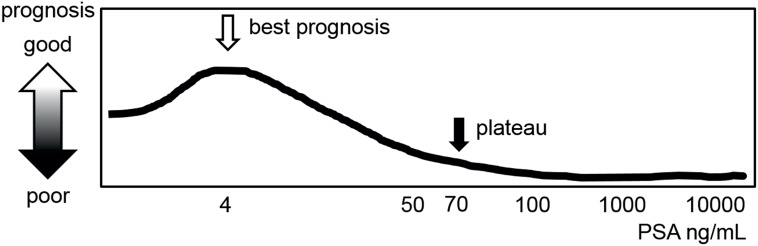
Schematic model of prognosis of patients with PCa based on initial PSA level. Previous studies suggested a role for initial PSA level as a prognostic marker when the PSA level is < 20 ng / mL and ≥ 100 ng / mL. This study revealed the relationship between PSA level and prognosis when the PSA level is between 20 and 100 ng / mL.

In our study, high T stage / high GS / the presence of metastasis shortened each prognosis and these results were consistent with previous studies (1, 2). Although PSA levels between 20 and 70 ng / mL were linearly associated with worse GS, higher prevalence of metastasis, and CRPC development (+), higher PSA levels between 70 and 100 ng / mL were not associated with poor OS, PCaSS, and CFS. Based on our previous studies and current results, we believe that when the PSA level reaches approximately 70 ng / mL prognosis might bottom and reach a plateau (Figure-3).

It is well known that the proliferation of PCa cells depends on the androgen / androgen receptor (AR) signal activity. Furthermore, PSA is an AR - regulated gene, and serum PSA levels reflect AR activity in PCa cells (5). However, we sometimes observed very aggressive PCa with a low PSA level and high GS (3). Pretreatment with total testosterone in patients with metastatic prostate disease has been investigated by many groups, as patients with PCa and low serum total testosterone levels have poor prognosis compared with those with PCa and high serum total testosterone levels (6-11). This suggests that androgen - AR signal - independent PCa might be of an aggressive nature with a high GS. AR expression level is downregulated by ADT, and other signals play key roles in the progression of PCa, particularly in invasion and metastasis (12, 13). Neither docetaxel nor cabazitaxel target androgen - AR signaling; nevertheless, they significantly prolong OS of patients with CRPC compared with that of the control group (14-16). Even in castration - sensitive patients with PCa, the addition of docetaxel to ADT improved survival compared with treatment with ADT alone (17). These results suggest the existence of androgen - AR signal - independent PCa cells, at least to some extent, in the early stage of PCa. In our study, although the percentage of patients with GS > 7 and the presence of metastasis increased between PSA levels 20 and 70 ng / mL significantly, there was no difference in the percentage of patients with GS > 7 and the presence of metastasis between PSA levels 70 and 100 ng / mL. The number of PCa cells which are dependent on AR signaling might not be important for patients with PSA ≥ 70 ng / mL, as indicated by the CFS rates (Figures 2E and F).

This study has a number of limitations. The small sample size may have prevented determination of the precise statistical significance of differences between groups. Larger prospective studies with longer follow-up periods and data from other ethnic backgrounds are needed to confirm our findings. Moreover, as a variety of treatments, including radical prostatectomy, radiation, and ADT were performed, some outcomes may not have been impacted only by PSA.

This is the first report showing PSA as a useful biomarker for predicting prognosis at levels between 20 and 70 ng / mL, whereas PSA could not be a prognostic factor in patients with PCa and PSA levels of ≥ 70 ng / mL. When the PSA level reaches approximately 70 ng / mL, prognosis might bottom and reach a plateau. These findings may help clinicians predict OS, PCaSS, and CFS rates and plan an appropriate treatment and follow-up schedule after diagnosis.

## ABBREVIATIONS

ADT: = androgen - deprivation therapy
CFS: = castration - resistant prostate cancer - free survival
CRPC: = castration - resistant prostate cancer
GS: = Gleason score
OS: = overall survival
PCa: = prostate cancer
PCaSS: = prostate cancer - specific survival
PSA: = prostate - specific antigen
